# Effect of self-assembled InAs islands on the interfacial roughness of optical-switched resonant tunneling diode

**DOI:** 10.1186/1556-276X-7-128

**Published:** 2012-02-14

**Authors:** Haitao Tian, Lu Wang, Zhenwu Shi, Huaiju Gao, Shuhui Zhang, Wenxin Wang, Hong Chen

**Affiliations:** 1 Beijing National Laboratory of Condensed Matter Physics, Institute of Physics, Chinese Academy of Sciences, No.8, 3rd South Street, Zhongguancun, Haidian District, Beijing, 100190, People's Republic of China; 2 Engineering Research Center of Solid-State Lighting, Department of Electrical Engineering and Automation, Tianjin Polytechnic University, No. 63 Chenglin Road, Hedong District, Tianjin, 300160, People's Republic of China; 3 Department of Materials Science and Technology, Harbin Institute of Technology, 92 West Dazhi Street, Nan Gang District, Harbin, 150001, People's Republic of China

**Keywords:** Quantum dots, high-resolution TEM, glancing incidence X-ray reflectivity, interface flatness, molecular beam epitaxy

## Abstract

Embedding a quantum dot [QD] layer between the double barriers of resonant tunneling diode [RTD] is proved to be an effective method to increase the sensitivity of QD-RTD single-photon detector. However, the interfacial flatness of this device would be worsened due to the introduction of quantum dots. In this paper, we demonstrate that the interfacial quality of this device can be optimized through increasing the growth temperature of AlAs up barrier. The glancing incidence X-ray reflectivity and the high-resolution transmission electron microscopy measurements show that the interfacial smoothness has been greatly improved, and the photo-luminescence test indicated that the InAs QDs were maintained at the same time. The smoother interface was attributed to the evaporation of segregated indium atoms at InGaAs surface layer.

**PACS:**

73.40.GK, 73.23._b, 73.21.La, 74.62.Dh

## Introduction

**Figure 1:**
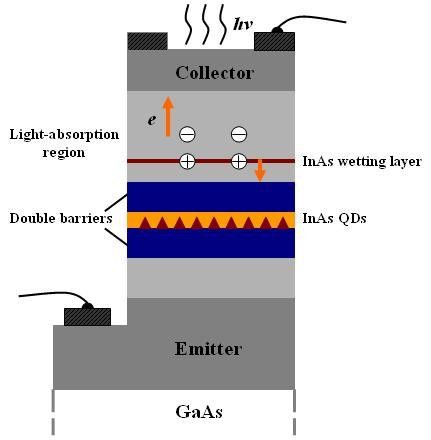
**Schematic of the device structure**.

Currently, it has been demonstrated that resonant tunneling diodes [RTD] containing a layer of self-assembled quantum dots [QD] could be used as high photo-excited carrier multiplication devices [1-4]. These detectors are based on sensing a change in the resonant tunneling current through the structure caused by the capture of a photo-excited hole in a quantum dot located near the active region of resonant tunneling diodes. However, in these devices, the modulate ability of the tunneling current through the resonant tunneling diode was relatively small. It is found that embedding the QD layer between the tunnel barriers can improve the multiplication factor; the schematic of this device structure is shown in Figure 1. For the coulomb potential energy of quantum dot to be greatly changed by nearby localized charge than quantum well, a much higher level of sensitivity (> 1,000%) and a means of opening/closing the current channel with a very high on/off ratio (> 50) were provided [5]. This structure expands prospects to further improve the performances of QD-RTD for charge-sensitive photon-counting detectors.

Nevertheless, if the quantum dot layer was built in the double barriers of RTD, the interfacial flatness may be worsened [6]. Because the interfacial flatness has a strong influence on the properties of RTD [7-9], the use of InAs QDs within double barriers may possibly deteriorate the performance of practical device. In this paper, it is shown that the use of QDs within AlAs barriers does deteriorate the interfacial quality, and then, this weakness was optimized through changing the growth condition. Detailed measurements show that the interfacial flatness has been greatly improved without dissolving the InAs QDs.

## Experimental details

All samples were grown by V80H solid-source molecular beam epitaxy system on GaAs semi-insulating (100) substrates, the growth rates were determined by the reflection high-energy electron diffraction oscillation technique. In order to investigate the effect of quantum dot layer on interface quality of QD-RTD, we prepared two samples which were assigned as RTD-1 and RTD-2, respectively. The growth process of RTD-2 can be described as follows: first, a 500-nm thick GaAs buffer layer was grown at 580°C, a 3-nm AlAs down barrier layer [DBL] was deposited afterwards at 610°C, and then the substrate temperature was lowered to 500°C. Subsequently, a 1-nm In_0.15_Ga_0.85_As-strained layer, a 1.6 mono-layer [ML] of InAs QDs, and a 4-nm In_0.15_Ga_0.85_As capping layer were grown, respectively, with the rate of 0.022 ML/s for InAs and 1.3 nm/s for the In_0.15_Ga_0.85_As layer. A 2-min growth interruption was introduced before the InAs layer growth and another 20 s after the formation of QDs. Finally, a 3-nm AlAs was deposited as the up barrier layer [UBL]. The structure of sample RTD-1 was the same as RTD-2 except that it did not contain an InAs QDs layer.

The glancing incidence X-ray reflectivity [GIXRR] was operated on a Bede D1 high-resolution triple-axis diffractometer (Bede Scientific Incorporated, 14 Inverness Drive East, Englewood, CO, USA). The high-resolution transmission electron microscopy [HRTEM] observation was conducted on a JEOL 2010 system (EM Lab Services, Inc., KA, USA). The photo-luminescence [PL] measurements were performed at 77 kelvin [K] using the 532-nm line. The resulting luminescence signal was analyzed with a grating mono-chromator and detected by a photon counting system.

## Results and discussion

**Figure 2:**
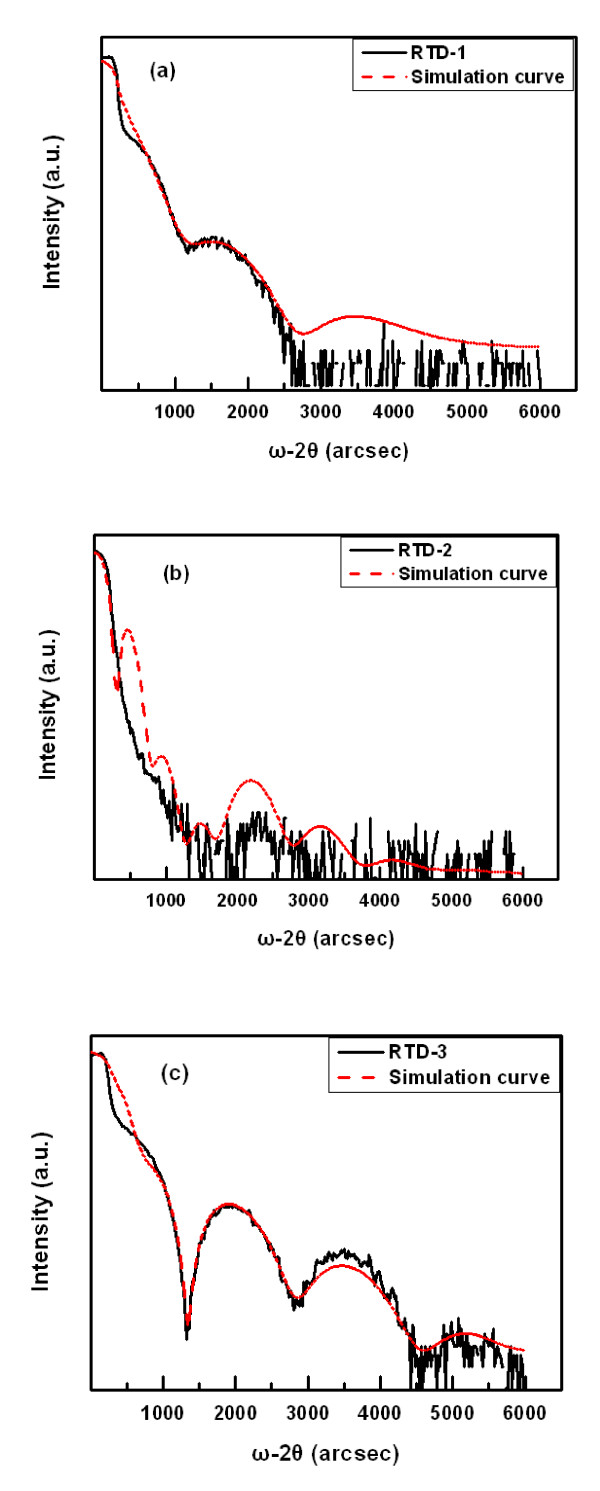
**GIXRR measurements of the experimental samples**. The GIXRR for (**a**) RTD-1, (**b**) RTD-2, and (**c**) RTD-3 are measured. GIXRR curve (black solid line) and corresponding simulation curve (red dash line)

In order to analyze the interface quality, GIXRR measurements have been carried out, and the experimental data were simulated with the commercial software of reflectivity and fluorescence simulation [RFS] provided by Bede (Bede Scientific Incorporated). The results are shown in Figure 2. Figure 2a,b shows the experimental curve and simulation curve of RTD-1 and RTD-2, respectively. A clear peak can be observed at the range of 1,000 to 2,000 arc sec in GIXRR curve of RTD-1, but nearly no satellite peak can be observed in Figure 2b which is RTD sample with embedded InAs QD layer. The appearance of the satellite peaks in Figure 2a suggests a better interfacial smoothness of RTD-1 than RTD-2. It is also shown in the same figure that the simulation curve is well-fitted with the experimental curve of the sample without InAs QD layer. Because the experimental curve of RTD-2 as shown in Figure 2b had no obvious satellite peaks, the simulation curve did not fit with the experimental curve accurately. The root mean square [RMS] roughness of different layers for the two samples were obtained and listed in Table 1. Note that due to the sharp decrease of the X-ray intensity during the measurement of RTD-2, the simulated curve cannot match the experimental results well. The RMS roughness of this sample is only listed as a reference but does not accurately represent the real structural characteristics of this sample. It can be clearly found that the RMS roughness of AlAs UBL was higher than DBL for both samples. Considerably, the same growth conditions were adopted for the two samples. There are reasons to believe that deposited InAs QDs between double barriers worsened the interfacial flatness.

**Table 1:** Simulated result of RMS roughness of every layer of RTD-1, RTD-2, and RTD-3

		AlAs DBL	InGaAs-strained layer	InAs QDs layer	InGaAs capping layer	AlAs UBL
RMS (nm)	RTD-1	0.15	0.19			0.31
	RTD-2	0.18	0.14	6.05	0.28	0.76
	RTD-3	0.17	0.15	4.62	0.17	0.18

DBL, down barrier layer; QD, quantum dot, UBL, up barrier layer; RMS, root mean square, RTD, resonant tunneling diode.

The penetration depth of X-ray is very low in the test of GIXRR, and the reflection light intensity will show a power exponent downward trend with the increase of penetration depth [10-12]. So, the GIXRR better reflects the quality of the interface close to the surface. Hence, it is believable that the decline of AlAs UBL/InGaAs interface flatness contributes to the deteriorative interfacial quality of RTD-2 more than AlAs DBL/InGaAs interface. Because the AlAs UBL/InGaAs hetero-junction was deposited after the growth of InAs QDs, it will definitely increase the difficulty to obtain a flat hetero-junction interface. Obviously, in order to put this type of QD-RTD into real use, the interfacial flatness must be improved.

To improve the interface quality, three methods may be feasible. The first one is depositing thicker InGaAs capping layer, but the increasing thickness of quantum well will introduce a serious degradation on resonant tunneling performance of RTD [13], at the same time, this change of material structure will increase the total strain accumulation of InAs/InGaAs system [14]. The second one is to raise the growth temperature of InGaAs capping layer. Higher temperature is conducive to increase the atom migration ability and, thus, improve the interface flatness. However, for the sake of the weaker In-As chemical bond, this approach may lead to the deviation of indium component of InGaAs capping layer from the setting value and may even cause the InAs QDs to dissolve. The last one is to increase the growth temperature of depositing AlAs UBL. Because the InGaAs layer is strained, the system tends to reduce the strain energy through segregating indium atoms onto surface [15,16]. This phenomenon will increase the roughness of InGaAs surface. Raising temperature after InGaAs growth as an annealing treatment can evaporate excess indium atoms at InGaAs layer surface and will result a better growth of front flatness [17,18]. So, we adopted the last method to grow simple RTD-3. Its structure was exactly the same as RTD-2; the only difference was the improved growth temperature of the AlAs UBL from 500°C to 610°C with an interruption of 2 min.

Figure 2c shows the GIXRR curve of RTD-3. The appearance of multi-level satellite peaks indicates that the interface quality has been truly improved. The experimental data were simulated with RFS, and the RMS roughness of every layer was also listed in Table 1. According to the simulation of RTD-3, the RMS roughness for InAs QDs layer was 4.62 nm which was consistent with the average height of the Stranski-Krastanov growth mode QDs (4 to 7 nm) [19,20], indicating that the simulating result was very close to real value; so, this simulation should be convincing. In Table 1, it is shown that the flatness of UBL has been greatly optimized via the improvement of growth condition. The RMS roughness of RTD-3 UBL decreased from 0.31 nm (of RTD-1) to 0.18 nm. In addition, it should also be noticed that UBL and DBL of RTD-3 have RMS roughness of 0.17 nm and 0.18 nm, respectively. Obviously, the interfacial flatness of UBL has been improved to be close to the level of DBL.

**Figure 3:**
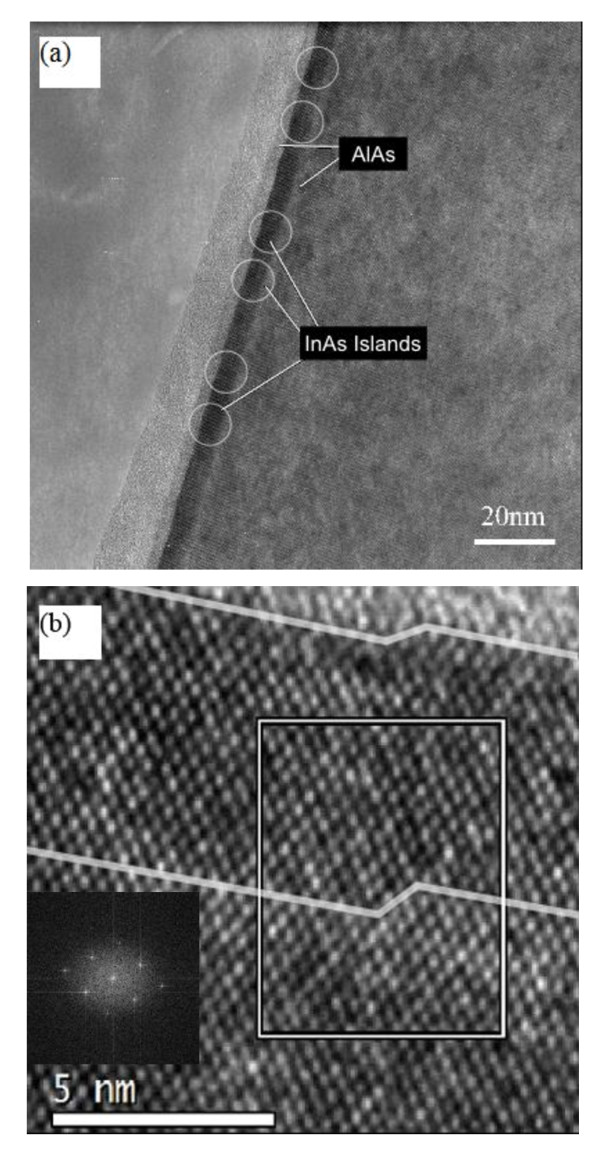
**HRTEM images for InAs/InGaAs/AlAs structure**. (**a**) Low magnification, and (**b**) high magnification. Inset image is the Fourier-transformed image of selected area.

In order to obtain the structural characteristics at the atomic level, the cross-sectional HRTEM image, which was taken along the [1 1 0] direction of sample RTD-3, was obtained. The results are shown in Figure 3. The position of the AlAs barriers and the InAs QDs was marked in Figure 3a, and 3b shows the enlarged image of the part. It is shown in Figure 3 that the roughness of AlAs UBL and DBL have no obvious difference and are both in the range of 1 to 3 ML. Another phenomenon could be observed from Figure 3b; both the interfaces of UBL and DBL have very similar 2 ML step at the same location. This feature suggests that, at least, parts of the steps in UBL were transferred from the DBL but did not form during the growth process of UBL. This phenomenon may also explain the same RMS of AlAs UBL and DBL measured by GIXRR.

**Figure 4:**
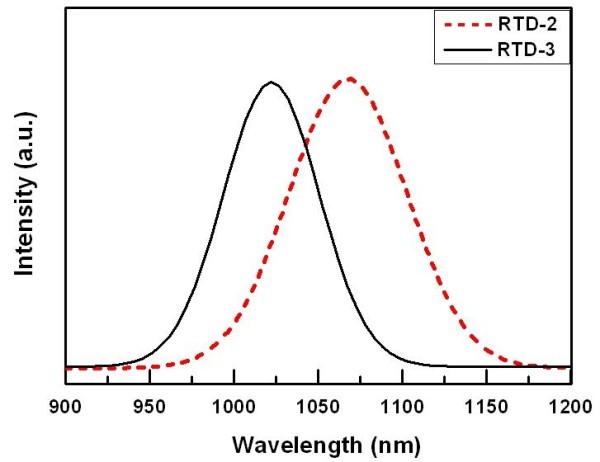
**PL spectra of RTD-2 (red dash line) and RTD-3 (black solid line) samples**.

Considering the change of growth condition that may dissolve InAs QDs, PL spectra at 77 K for samples RTD-2 and RTD-3 were acquired and shown in Figure 4. It can be found that each PL spectrum shows a dominant peak for the two samples, respectively, which is related to the inter-band transitions of InAs QDs. This result indicated that using higher temperature for UBL cannot dissolve InAs QDs. In addition, the PL peak position of sample RTD-3 shifts to shorter wavelengths of 27 nm (from 1,051 nm to 1,024 nm). This behavior may be attributed to the lower indium composition of InAs QDs resulting from local inter-diffusion of In and Ga atoms in the InGaAs/InAs QDs layers when using higher growth temperature [21].

## Conclusions

We have studied the interface quality of QD-RTD with a novel structure of InAs QDs incorporated in the double barriers of RTD. GIXRR was employed to test the roughness of InGaAs/AlAs hetero-junction interfaces. It is found that the interfacial flatness was positively deteriorated due to the deposition of InAs QDs layer. In order to optimized this defect, higher growth temperature was used in the growth of AlAs UBL. GIXRR measurement shows that the interfacial flatness of UBL has been improved to be close to the level of DBL, and subsequently, this result was verificated by HRTEM test. Meanwhile, PL measurement demonstrates that the InAs QDs were well maintained after the changing of growth condition. The improving quality of interface that could be ascribed to annealing treatment can evaporate excess indium atoms of InGaAs layer surface which resulted from indium segregation. This result could be used to further improve the performances of this potential structure of QD-RTD.

## Competing interests

The authors declare that they have no competing interests.

## Authors' contributions

HT participated in the MBE growth, carried out the GIXRR measurements, and drafted the manuscript. SZ conducted the STEM and PL measurements. ZS and HG conducted the MBE growth. WW and HC coordinated the study. LW provided the idea and supervised the study. All authors read and approved the final manuscript.
